# μ-Hexa­thio­metadiphosphato-bis­[(1,4,7,10,13,16-hexa­oxa­cyclo­octa­decane-κ^6^ *O*)rubidium] aceto­nitrile disolvate

**DOI:** 10.1107/S1600536813032121

**Published:** 2013-11-30

**Authors:** Mimoza Gjikaj, Niels-Patrick Pook, Flora Qarri

**Affiliations:** a Institute of Inorganic and Analytical Chemistry, Clausthal University of Technology, Paul-Ernst-Strasse 4, D-38678 Clausthal-Zellerfeld, Germany; b Chemistry Department, University of Vlora, Sheshi Pavaresia, 9401 Vlore, Albania

## Abstract

The asymmetric unit of the title compound, [Rb_2_(P_2_S_6_)(C_12_H_24_O_6_)_2_]·2CH_3_CN, contains one half of an [Rb(18-crown-6)_2_]_2_[P_2_S_6_] unit and one aceto­nitrile solvent mol­ecule. The [Rb(18-crown-6)]_2_[P_2_S_6_] unit is completed by inversion symmetry. Its Rb^+^ ion is situated near the centre of the macrocyclic cavity, but is displaced by 0.8972 (1) Å from the O atoms of the crown in the direction of the [P_2_S_6_]^2−^ moiety. The overall coordination number of the cation is eight, defined by the six crown ether O atoms and by two terminal S atoms of the [P_2_S_6_]^2−^ anion. The hexa­thio­metadiphosphate anion is built up from two tetra­hedral PS_4_ units joined together by a common edge. The crystal structure is characterized by alternating layers of [Rb(18-crown-6)]_2_[P_2_S_6_] and aceto­nitrile solvent mol­ecules stacked along [010].

## Related literature

For the synthesis of hexa­thio­metadiphosphates, see: Thilo & Ladwig (1962 ▶). For the crystal structures of hexa­thio­metadiphosphates, see: Toffoli *et al.* (1978 ▶); Brockner *et al.* (1985 ▶). For the crystal structures of alkali crown ether hexa­thio­metadiphosphates, see: Gjikaj *et al.* (2005 ▶, 2006 ▶).
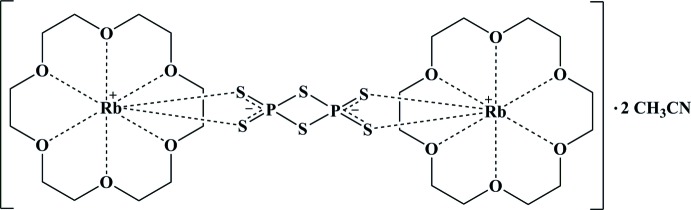


## Experimental

### 

#### Crystal data

[Rb_2_(P_2_S_6_)(C_12_H_24_O_6_)_2_]·2C_2_H_3_N
*M* *_r_* = 1035.98
Monoclinic, 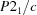

*a* = 8.2261 (9) Å
*b* = 17.1054 (15) Å
*c* = 16.5895 (18) Å
β = 95.520 (9)°
*V* = 2323.5 (4) Å^3^
*Z* = 2
Mo *K*α radiation
μ = 2.50 mm^−1^
*T* = 223 K
0.29 × 0.26 × 0.22 mm

#### Data collection

Stoe IPDSII diffractometer
Absorption correction: numerical (*X-SHAPE* and *X-RED*; Stoe & Cie, 1999 ▶, 2001 ▶) *T*_min_ = 0.490, *T*_max_ = 0.577
26056 measured reflections
4400 independent reflections
3450 reflections with *I* > 2σ(*I*)
*R*_int_ = 0.092

#### Refinement

*R*[*F* ^2^ > 2σ(*F* ^2^)] = 0.047
*wR*(*F* ^2^) = 0.074
*S* = 1.15
4400 reflections
343 parameters
All H-atom parameters refined
Δρ_max_ = 0.50 e Å^−3^
Δρ_min_ = −0.28 e Å^−3^

### 

Data collection: *X-AREA* (Stoe & Cie, 2002 ▶); cell refinement: *X-AREA*; data reduction: *X-AREA*; program(s) used to solve structure: *SHELXS97* (Sheldrick, 2008 ▶); program(s) used to refine structure: *SHELXL97* (Sheldrick, 2008 ▶); molecular graphics: *ORTEP-3 for Windows* (Farrugia, 2012 ▶); software used to prepare material for publication: *SHELXL97*, *PLATON* (Spek, 2009 ▶) and *publCIF* (Westrip, 2010 ▶).

## Supplementary Material

Crystal structure: contains datablock(s) I. DOI: 10.1107/S1600536813032121/wm2785sup1.cif

Structure factors: contains datablock(s) I. DOI: 10.1107/S1600536813032121/wm2785Isup2.hkl

Supplementary material file. DOI: 10.1107/S1600536813032121/wm2785Isup3.cdx

Additional supplementary materials:  crystallographic information; 3D view; checkCIF report

## Figures and Tables

**Fig. 1.**
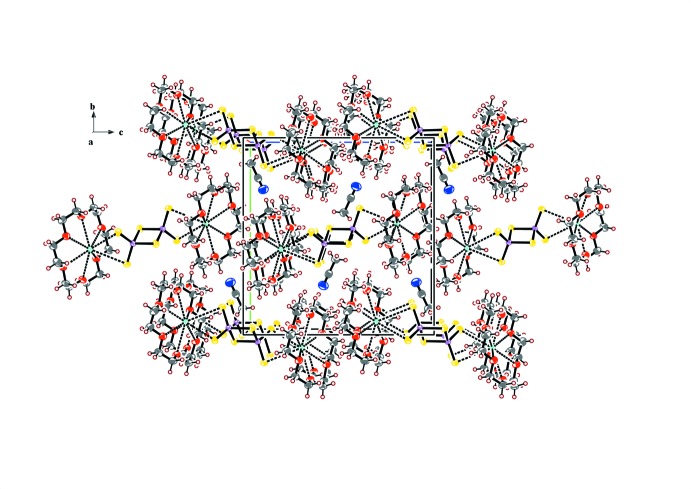
The crystal structure of the title compound. Displacement ellipsoids at the 50% probability level. The Rb—O and Rb—S bonds are shown as dashed lines.

**Fig. 2.**
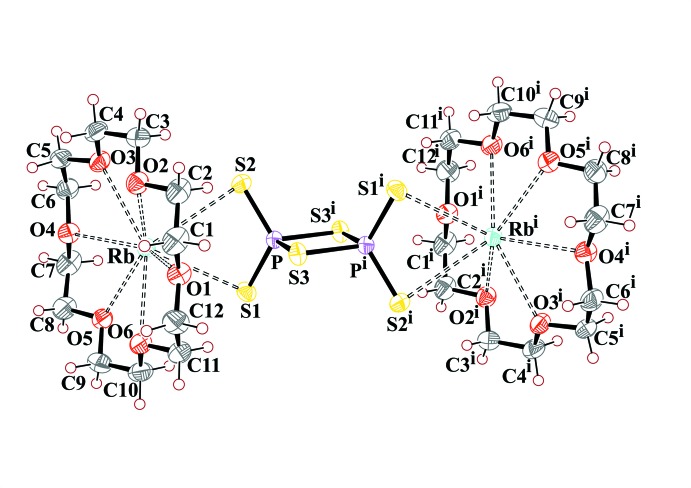
The molecular structure of the title compound with atom labels and 50% probability displacement ellipsoids. The Rb—O and Rb—S bonds are shown as dashed lines. [Symmetry codes: (i) -x, -y + 1, -z - 1.]

## supplementary crystallographic information

### 1. Comment

The first thio­phosphates with empirical formula *M*PS_3_ (*M* = Na, Ag and Tl), were described by Thilo & Ladwig (1962). However, the crystal structure determination of Ag_2_P_2_S_6_ proved the presence of isolated P_2_S_6_^2-^ anions (Toffoli *et al.*, 1978). The first alkali metal hexa­thio­metadiphosphate structures, *M*_2_P_2_S_6_ (*M* = K and Cs), were determined by Brockner *et al.* (1985). By using crown ether as complexing agent, alkali hexa­thio­metadiphosphates, *M*_2_P_2_S_6_ (*M* = Na and K), can be dissolved in CH_3_CN. Such crown-ether-stabilized thio­phosphates were obtained in crystalline form by Gjikaj *et al.* (2005, 2006).

The structure of the title compound, [Rb(18-crown-6)]_2_[P_2_S_6_]^.^2CH_3_CN, is isotypic with [K(18-crown-6)]_2_[P_2_S_6_]^.^2CH_3_CN (Gjikaj *et al.*, 2005) and is characterized by alternating layers of [Rb(18-crown-6)]_2_[P_2_S_6_] and aceto­nitrile molecules stacked along [010]. The asymmetric unit consists of one half of an [Rb(18-crown-6)]_2_[P_2_S_6_] unit, which is located on a centre of inversion, and is completed by one aceto­nitrile molecule. The eightfold coordination environment of rubidium is defined by the six crown ether oxygen atoms and by two terminal sulfur atoms of the hexa­thio­metadiphosphate anion (Fig. 2). The hexa­thio­metadiphosphate anion is built up by two edge-sharing PS_4_ units, each with a tetra­hedral arrangement. The P–S bond lengths are ranging from 1.9630 (13) to 2.1419 (13) Å. All bond lengths and angles are comparable to those found for [K(18-crown-6)]_2_[P_2_S_6_]^.^2CH_3_CN (Gjikaj *et al.*, 2005).

### 2. Experimental

Rubidium hexa­thio­metadiphosphate was prepared by high-temperature element synthesis using the procedure reported by Brockner *et al.* (1985). A solution of bis­[(1,4,7,10,13,16-hexaoxa­cyclo­octa­decane-*κ*^6^O)rubidium] hexa­thio­metadiphosphate was obtained by adding rubidium hexa­thio­metadiphosphate to a solution of 18-crown-6 in dry aceto­nitrile.

### 2.1. Refinement

All hydrogen atoms were located in a difference Fourier map and were refined isotropically with no restraints.

### Crystal data

**Table:**

[Rb_2_(P_2_S_6_)(C_12_H_24_O_6_)_2_]·2C_2_H_3_N	*F*(000) = 1064
*M**_r_* = 1035.98	block, yellow
Monoclinic, *P*2_1_/*c*	*D*_x_ = 1.481 Mg m^−^^3^
Hall symbol: -P 2yn	Mo *K*α radiation, λ = 0.71073 Å
*a* = 8.2261 (9) Å	Cell parameters from 41621 reflections
*b* = 17.1054 (15) Å	θ = 2.4–25.7°
*c* = 16.5895 (18) Å	µ = 2.50 mm^−^^1^
β = 95.520 (9)°	*T* = 223 K
*V* = 2323.5 (4) Å^3^	Block, yellow
*Z* = 2	0.29 × 0.26 × 0.22 mm

### Data collection

**Table:**

Stoe IPDSII diffractometer	4400 independent reflections
Radiation source: fine-focus sealed tube	3450 reflections with *I* > 2σ(*I*)
Graphite monochromator	*R*_int_ = 0.092
ω–scans	θ_max_ = 25.7°, θ_min_ = 2.4°
Absorption correction: numerical (*X-SHAPE* and *X-RED*; Stoe & Cie, 1999, 2001)	*h* = −10→9
*T*_min_ = 0.490, *T*_max_ = 0.577	*k* = −20→20
26056 measured reflections	*l* = −20→20

### Refinement

**Table:**

Refinement on *F*^2^	Primary atom site location: structure-invariant direct methods
Least-squares matrix: full	Secondary atom site location: difference Fourier map
*R*[*F*^2^ > 2σ(*F*^2^)] = 0.047	Hydrogen site location: inferred from neighbouring sites
*wR*(*F*^2^) = 0.074	All H-atom parameters refined
*S* = 1.15	*w* = 1/[σ^2^(*F*_o_^2^) + (0.0188*P*)^2^ + 1.1961*P*] where *P* = (*F*_o_^2^ + 2*F*_c_^2^)/3
4400 reflections	(Δ/σ)_max_ < 0.001
343 parameters	Δρ_max_ = 0.50 e Å^−^^3^
0 restraints	Δρ_min_ = −0.28 e Å^−^^3^

### Special details

**Table:**

Geometry. All e.s.d.'s (except the e.s.d. in the dihedral angle between two l.s. planes) are estimated using the full covariance matrix. The cell e.s.d.'s are taken into account individually in the estimation of e.s.d.'s in distances, angles and torsion angles; correlations between e.s.d.'s in cell parameters are only used when they are defined by crystal symmetry. An approximate (isotropic) treatment of cell e.s.d.'s is used for estimating e.s.d.'s involving l.s. planes.
Refinement. Refinement of *F*^2^ against ALL reflections. The weighted *R*-factor *wR* and goodness of fit *S* are based on *F*^2^, conventional *R*-factors *R* are based on *F*, with *F* set to zero for negative *F*^2^. The threshold expression of *F*^2^ > σ(*F*^2^) is used only for calculating *R*-factors(gt) *etc*. and is not relevant to the choice of reflections for refinement. *R*-factors based on *F*^2^ are statistically about twice as large as those based on *F*, and *R*- factors based on ALL data will be even larger.

### Fractional atomic coordinates and isotropic or equivalent isotropic displacement parameters (Å^2^)

**Table:**

	*x*	*y*	*z*	*U*_iso_*/*U*_eq_	
Rb	0.01688 (5)	0.43425 (2)	−0.19589 (2)	0.03401 (10)	
S1	0.01382 (16)	0.35946 (6)	−0.38945 (6)	0.0451 (3)	
S2	−0.20728 (13)	0.51995 (6)	−0.36048 (6)	0.0374 (2)	
S3	0.14602 (13)	0.53670 (6)	−0.44812 (5)	0.0348 (2)	
P	−0.05739 (12)	0.46416 (5)	−0.42619 (5)	0.0290 (2)	
O1	0.2674 (4)	0.54136 (15)	−0.23640 (16)	0.0410 (6)	
O2	0.0200 (4)	0.60293 (16)	−0.14890 (16)	0.0415 (7)	
O3	−0.2169 (4)	0.50147 (15)	−0.09188 (15)	0.0406 (6)	
O4	−0.1161 (3)	0.34506 (15)	−0.06497 (16)	0.0394 (6)	
O5	0.1325 (4)	0.27789 (15)	−0.14684 (15)	0.0412 (7)	
O6	0.3663 (4)	0.38439 (16)	−0.19993 (16)	0.0416 (7)	
C1	0.2628 (7)	0.6212 (3)	−0.2123 (3)	0.0509 (12)	
C2	0.0893 (7)	0.6461 (3)	−0.2104 (3)	0.0482 (11)	
C3	−0.1428 (7)	0.6269 (3)	−0.1389 (3)	0.0499 (11)	
C4	−0.2044 (7)	0.5824 (3)	−0.0707 (3)	0.0479 (11)	
C5	−0.2661 (7)	0.4551 (3)	−0.0274 (3)	0.0525 (12)	
C6	−0.2744 (6)	0.3710 (3)	−0.0527 (3)	0.0540 (12)	
C7	−0.1060 (6)	0.2626 (2)	−0.0796 (3)	0.0473 (11)	
C8	0.0674 (6)	0.2404 (2)	−0.0800 (3)	0.0435 (10)	
C9	0.3003 (6)	0.2593 (3)	−0.1507 (3)	0.0457 (10)	
C10	0.3625 (7)	0.3033 (3)	−0.2188 (3)	0.0476 (11)	
C11	0.4266 (6)	0.4296 (3)	−0.2630 (3)	0.0499 (11)	
C12	0.4300 (6)	0.5138 (3)	−0.2389 (3)	0.0513 (11)	
N13	−0.5159 (6)	0.2422 (3)	−0.4116 (3)	0.0750 (13)	
C14	−0.5164 (6)	0.3022 (3)	−0.4407 (3)	0.0478 (10)	
C15	−0.5201 (8)	0.3788 (3)	−0.4778 (4)	0.0580 (13)	
H1A	0.324 (6)	0.633 (3)	−0.160 (3)	0.054 (13)*	
H1B	0.323 (7)	0.652 (3)	−0.252 (3)	0.073 (16)*	
H2A	0.026 (5)	0.639 (2)	−0.261 (3)	0.044 (12)*	
H2B	0.098 (6)	0.702 (3)	−0.197 (3)	0.062 (14)*	
H3A	−0.146 (6)	0.683 (3)	−0.125 (3)	0.065 (14)*	
H3B	−0.212 (6)	0.618 (2)	−0.192 (3)	0.050 (13)*	
H4A	−0.303 (6)	0.603 (3)	−0.056 (3)	0.054 (13)*	
H4B	−0.129 (6)	0.590 (3)	−0.018 (3)	0.056 (13)*	
H5A	−0.373 (7)	0.473 (3)	−0.016 (3)	0.077 (17)*	
H5B	−0.179 (6)	0.463 (2)	0.019 (3)	0.052 (13)*	
H6A	−0.308 (5)	0.342 (2)	−0.008 (3)	0.047 (12)*	
H6B	−0.341 (6)	0.367 (3)	−0.106 (3)	0.062 (15)*	
H7A	−0.143 (5)	0.237 (2)	−0.036 (2)	0.041 (11)*	
H7B	−0.179 (7)	0.253 (3)	−0.132 (3)	0.077 (17)*	
H8A	0.078 (5)	0.189 (3)	−0.083 (2)	0.041 (11)*	
H8B	0.137 (5)	0.259 (2)	−0.027 (3)	0.050 (12)*	
H9A	0.319 (6)	0.204 (3)	−0.158 (3)	0.056 (13)*	
H9B	0.364 (5)	0.271 (2)	−0.097 (3)	0.046 (12)*	
H10A	0.300 (6)	0.296 (3)	−0.267 (3)	0.050 (13)*	
H10B	0.470 (6)	0.287 (3)	−0.229 (3)	0.054 (13)*	
H11A	0.537 (6)	0.413 (3)	−0.269 (3)	0.054 (13)*	
H11B	0.353 (6)	0.420 (3)	−0.314 (3)	0.061 (14)*	
H12A	0.485 (6)	0.520 (3)	−0.186 (3)	0.052 (13)*	
H12B	0.480 (6)	0.543 (2)	−0.282 (3)	0.051 (13)*	
H15A	−0.451 (8)	0.411 (4)	−0.446 (4)	0.09 (2)*	
H15B	−0.493 (7)	0.374 (3)	−0.531 (4)	0.083 (18)*	
H15C	−0.630 (9)	0.396 (4)	−0.485 (4)	0.11 (3)*	

### Atomic displacement parameters (Å^2^)

**Table:**

	*U*^11^	*U*^22^	*U*^33^	*U*^12^	*U*^13^	*U*^23^
Rb	0.0386 (2)	0.03336 (18)	0.03045 (17)	−0.00168 (18)	0.00526 (13)	0.00247 (16)
S1	0.0684 (8)	0.0288 (5)	0.0373 (5)	0.0062 (5)	0.0010 (5)	0.0021 (4)
S2	0.0354 (6)	0.0432 (5)	0.0349 (5)	0.0033 (4)	0.0099 (4)	−0.0027 (4)
S3	0.0333 (5)	0.0429 (5)	0.0282 (4)	−0.0058 (4)	0.0022 (4)	0.0005 (4)
P	0.0323 (6)	0.0286 (4)	0.0263 (4)	0.0002 (4)	0.0043 (4)	0.0010 (4)
O1	0.0347 (16)	0.0398 (15)	0.0487 (15)	−0.0043 (12)	0.0055 (12)	0.0030 (12)
O2	0.0426 (18)	0.0380 (15)	0.0440 (15)	0.0020 (13)	0.0046 (13)	0.0031 (12)
O3	0.0415 (17)	0.0390 (15)	0.0412 (15)	0.0022 (13)	0.0036 (12)	0.0018 (12)
O4	0.0361 (17)	0.0375 (15)	0.0453 (15)	−0.0053 (12)	0.0072 (12)	0.0027 (12)
O5	0.0460 (18)	0.0349 (14)	0.0427 (15)	0.0005 (13)	0.0048 (13)	0.0079 (11)
O6	0.0401 (17)	0.0418 (15)	0.0433 (15)	−0.0003 (13)	0.0063 (13)	0.0048 (12)
C1	0.058 (3)	0.038 (2)	0.058 (3)	−0.017 (2)	0.013 (2)	−0.002 (2)
C2	0.063 (3)	0.034 (2)	0.048 (3)	−0.003 (2)	0.008 (2)	0.0046 (19)
C3	0.056 (3)	0.034 (2)	0.059 (3)	0.005 (2)	0.003 (2)	−0.001 (2)
C4	0.045 (3)	0.047 (3)	0.053 (3)	0.005 (2)	0.008 (2)	−0.0113 (19)
C5	0.045 (3)	0.059 (3)	0.057 (3)	0.011 (2)	0.023 (2)	0.011 (2)
C6	0.036 (3)	0.060 (3)	0.068 (3)	−0.004 (2)	0.016 (2)	0.019 (3)
C7	0.059 (3)	0.033 (2)	0.050 (3)	−0.010 (2)	0.007 (2)	0.0054 (19)
C8	0.058 (3)	0.027 (2)	0.046 (2)	−0.002 (2)	0.009 (2)	0.0046 (17)
C9	0.047 (3)	0.040 (2)	0.050 (3)	0.011 (2)	0.002 (2)	0.0071 (19)
C10	0.052 (3)	0.042 (2)	0.050 (3)	0.010 (2)	0.009 (2)	−0.0009 (19)
C11	0.037 (3)	0.057 (3)	0.057 (3)	0.004 (2)	0.014 (2)	0.015 (2)
C12	0.035 (3)	0.056 (3)	0.064 (3)	−0.008 (2)	0.009 (2)	0.014 (2)
N13	0.086 (4)	0.058 (3)	0.081 (3)	0.012 (3)	0.011 (3)	0.009 (2)
C14	0.043 (3)	0.054 (3)	0.046 (2)	0.006 (2)	0.003 (2)	−0.008 (2)
C15	0.065 (4)	0.050 (3)	0.058 (3)	−0.010 (3)	−0.002 (3)	0.003 (2)

### Geometric parameters (Å, º)

**Table:**

Rb—O1	2.885 (3)	C3—C4	1.491 (7)
Rb—O5	2.928 (3)	C3—H3A	0.98 (5)
Rb—O3	2.939 (3)	C3—H3B	1.01 (5)
Rb—O4	2.949 (3)	C4—H4A	0.93 (5)
Rb—O2	2.988 (3)	C4—H4B	1.02 (5)
Rb—O6	3.005 (3)	C5—C6	1.499 (7)
Rb—S1	3.4544 (11)	C5—H5A	0.97 (6)
Rb—S2	3.4692 (11)	C5—H5B	1.01 (5)
S1—P	1.9630 (13)	C6—H6A	0.96 (4)
S2—P	1.9692 (13)	C6—H6B	1.00 (5)
S3—P^i^	2.1418 (13)	C7—C8	1.477 (7)
S3—P	2.1421 (14)	C7—H7A	0.92 (4)
P—S3^i^	2.1419 (13)	C7—H7B	1.02 (5)
O1—C12	1.423 (6)	C8—H8A	0.89 (4)
O1—C1	1.424 (5)	C8—H8B	1.05 (4)
O2—C2	1.422 (5)	C9—C10	1.489 (6)
O2—C3	1.425 (6)	C9—H9A	0.97 (5)
O3—C5	1.421 (5)	C9—H9B	1.01 (4)
O3—C4	1.430 (5)	C10—H10A	0.91 (5)
O4—C6	1.409 (5)	C10—H10B	0.96 (5)
O4—C7	1.435 (5)	C11—C12	1.495 (7)
O5—C9	1.423 (5)	C11—H11A	0.97 (5)
O5—C8	1.428 (5)	C11—H11B	1.01 (5)
O6—C10	1.422 (5)	C12—H12A	0.95 (5)
O6—C11	1.428 (5)	C12—H12B	1.00 (4)
C1—C2	1.493 (7)	N13—C14	1.135 (6)
C1—H1A	0.98 (5)	C14—C15	1.446 (7)
C1—H1B	1.01 (5)	C15—H15A	0.92 (7)
C2—H2A	0.96 (4)	C15—H15B	0.93 (6)
C2—H2B	0.97 (5)	C15—H15C	0.95 (7)
			
O1—Rb—O5	115.13 (8)	C4—C3—H3A	108 (3)
O1—Rb—O3	114.25 (8)	Rb—C3—H3A	160 (3)
O5—Rb—O3	114.06 (7)	O2—C3—H3B	108 (3)
O1—Rb—O4	145.48 (8)	C4—C3—H3B	113 (3)
O5—Rb—O4	57.07 (8)	Rb—C3—H3B	80 (2)
O3—Rb—O4	57.31 (7)	H3A—C3—H3B	108 (4)
O1—Rb—O2	57.26 (8)	O3—C4—C3	109.2 (4)
O5—Rb—O2	144.60 (8)	O3—C4—H4A	112 (3)
O3—Rb—O2	57.22 (8)	C3—C4—H4A	112 (3)
O4—Rb—O2	107.56 (7)	O3—C4—H4B	111 (3)
O1—Rb—O6	57.92 (8)	C3—C4—H4B	111 (3)
O5—Rb—O6	57.38 (8)	H4A—C4—H4B	102 (4)
O3—Rb—O6	143.65 (8)	O3—C5—C6	109.5 (4)
O4—Rb—O6	107.03 (7)	O3—C5—H5A	108 (3)
O2—Rb—O6	107.20 (8)	C6—C5—H5A	110 (3)
O1—Rb—S1	87.72 (6)	O3—C5—H5B	105 (3)
O5—Rb—S1	83.87 (5)	C6—C5—H5B	111 (3)
O3—Rb—S1	138.08 (6)	H5A—C5—H5B	113 (4)
O4—Rb—S1	121.53 (6)	O4—C6—C5	109.0 (4)
O2—Rb—S1	126.82 (5)	O4—C6—H6A	107 (3)
O6—Rb—S1	78.21 (6)	C5—C6—H6A	107 (3)
O1—Rb—S2	83.44 (6)	Rb—C6—H6A	155 (3)
O5—Rb—S2	137.62 (6)	O4—C6—H6B	107 (3)
O3—Rb—S2	88.20 (6)	C5—C6—H6B	109 (3)
O4—Rb—S2	126.26 (6)	Rb—C6—H6B	78 (3)
O2—Rb—S2	77.75 (6)	H6A—C6—H6B	118 (4)
O6—Rb—S2	122.65 (5)	O4—C7—C8	108.9 (4)
S1—Rb—S2	57.93 (3)	O4—C7—H7A	108 (3)
P—S1—Rb	85.75 (4)	C8—C7—H7A	106 (3)
P—S2—Rb	85.25 (4)	Rb—C7—H7A	156 (3)
P^i^—S3—P	87.88 (5)	O4—C7—H7B	105 (3)
S1—P—S2	117.02 (6)	C8—C7—H7B	116 (3)
S1—P—S3^i^	111.07 (6)	Rb—C7—H7B	82 (3)
S2—P—S3^i^	111.69 (6)	H7A—C7—H7B	112 (4)
S1—P—S3	111.50 (7)	O5—C8—C7	108.9 (4)
S2—P—S3	110.75 (6)	O5—C8—H8A	111 (3)
S3^i^—P—S3	92.12 (5)	C7—C8—H8A	111 (3)
S1—P—Rb	63.64 (4)	O5—C8—H8B	108 (2)
S2—P—Rb	64.06 (4)	C7—C8—H8B	111 (2)
S3^i^—P—Rb	166.70 (5)	H8A—C8—H8B	107 (3)
S3—P—Rb	101.17 (4)	O5—C9—C10	109.1 (4)
C12—O1—C1	112.1 (4)	O5—C9—H9A	113 (3)
C12—O1—Rb	119.4 (2)	C10—C9—H9A	110 (3)
C1—O1—Rb	120.3 (3)	O5—C9—H9B	110 (2)
C2—O2—C3	112.3 (3)	C10—C9—H9B	112 (2)
C2—O2—Rb	108.0 (2)	H9A—C9—H9B	103 (4)
C3—O2—Rb	108.9 (2)	O6—C10—C9	109.1 (4)
C5—O3—C4	112.0 (3)	O6—C10—H10A	109 (3)
C5—O3—Rb	118.3 (2)	C9—C10—H10A	113 (3)
C4—O3—Rb	119.2 (2)	Rb—C10—H10A	78 (3)
C6—O4—C7	113.8 (3)	O6—C10—H10B	109 (3)
C6—O4—Rb	110.9 (2)	C9—C10—H10B	112 (3)
C7—O4—Rb	110.6 (2)	Rb—C10—H10B	160 (3)
C9—O5—C8	111.9 (3)	H10A—C10—H10B	105 (4)
C9—O5—Rb	118.8 (2)	O6—C11—C12	108.9 (4)
C8—O5—Rb	119.3 (2)	O6—C11—H11A	108 (3)
C10—O6—C11	111.6 (3)	C12—C11—H11A	109 (3)
C10—O6—Rb	106.3 (3)	Rb—C11—H11A	160 (3)
C11—O6—Rb	105.3 (2)	O6—C11—H11B	108 (3)
O1—C1—C2	109.4 (4)	C12—C11—H11B	112 (3)
O1—C1—H1A	115 (3)	Rb—C11—H11B	76 (3)
C2—C1—H1A	109 (3)	H11A—C11—H11B	111 (4)
O1—C1—H1B	107 (3)	O1—C12—C11	109.5 (4)
C2—C1—H1B	113 (3)	O1—C12—H12A	108 (3)
H1A—C1—H1B	103 (4)	C11—C12—H12A	110 (3)
O2—C2—C1	108.5 (4)	O1—C12—H12B	108 (3)
O2—C2—H2A	110 (3)	C11—C12—H12B	107 (2)
C1—C2—H2A	113 (3)	H12A—C12—H12B	115 (4)
Rb—C2—H2A	82 (2)	N13—C14—C15	179.0 (6)
O2—C2—H2B	112 (3)	C14—C15—H15A	108 (4)
C1—C2—H2B	103 (3)	C14—C15—H15B	109 (4)
Rb—C2—H2B	162 (3)	H15A—C15—H15B	114 (5)
H2A—C2—H2B	110 (4)	C14—C15—H15C	109 (4)
O2—C3—C4	109.5 (4)	H15A—C15—H15C	115 (6)
O2—C3—H3A	111 (3)	H15B—C15—H15C	102 (5)

Symmetry code: (i) −*x*, −*y*+1, −*z*−1.
